# Combining cell-free DNA fragmentomes and total tumour volume improves prognostication and tumour response evaluation in patients with colorectal cancer liver metastases

**DOI:** 10.1016/j.ebiom.2025.106081

**Published:** 2025-12-16

**Authors:** Nerma Crnovrsanin, J. Michiel Zeeuw, Mahsoem Ali, Ruby Kemna, Bahar Alipanahi, Keith Lumbard, Zachary L. Skidmore, Lorenzo Rinaldi, Iris van 't Erve, Nina J. Wesdorp, Joost Huiskens, Denise van Steijn, Jan Hein van Waesberghe, Janneke van den Bergh, Irena Nota, Shira Moos, Marinde J.G. Bond, Lana Meiqari, Iris Huitink, Elisa Giovannetti, Jaap Stoker, Inez Verpalen, Daan van den Broek, Gerrit A. Meijer, Rutger-Jan Swijnenburg, Cornelis J.A. Punt, Robert B. Scharpf, Alessandro Leal, Nicholas C. Dracopoli, Victor E. Velculescu, Niels F.M. Kok, Geert Kazemier, Remond J.A. Fijneman

**Affiliations:** a Department of Pathology, Netherlands Cancer Institute, Amsterdam, the Netherlands; b Department of General, Abdominal and Transplantation Surgery, University Hospital Heidelberg, Heidelberg, Germany; c Department of Surgery, Amsterdam UMC, Vrije Universiteit Amsterdam, Amsterdam, the Netherlands; d Cancer Center Amsterdam, Amsterdam, the Netherlands; e Delfi Diagnostics, Inc., Baltimore, MD, USA; f Department of Radiology and Nuclear Medicine, Amsterdam UMC, Vrije Universiteit Amsterdam, Amsterdam, the Netherlands; g Department of Epidemiology, Julius Center for Health Sciences and Primary Care, University Medical Center Utrecht, Utrecht, the Netherlands; h The Sidney Kimmel Comprehensive Cancer Center, Johns Hopkins University School of Medicine, Baltimore, MD, USA; i Department of Radiology and Nuclear Medicine, Amsterdam UMC, University of Amsterdam, Amsterdam, the Netherlands; j Department of Laboratory Medicine, The Netherlands Cancer Institute, Amsterdam, the Netherlands; k Department of Surgery, Netherlands Cancer Institute, Amsterdam, the Netherlands

**Keywords:** Colorectal liver metastases, Prognosis, Liquid biopsies, Cell-free DNA fragmentome, Total tumour volume, Risk stratification, Treatment response

## Abstract

**Background:**

Treatment decisions in patients with unresectable colorectal liver metastases (CRLM) are largely guided by radiological response to induction systemic therapy. However, radiological assessment alone provides an imprecise estimate of underlying tumour biology or treatment response. Circulating tumour DNA (ctDNA) is an emerging biomarker that can support clinical decision-making. This study evaluated the independent prognostic value of radiological tumour burden and DELFI-TF, a tumour tissue- and mutation-independent cell-free DNA (cfDNA) fragmentome-based ctDNA assay.

**Methods:**

We analysed 202 plasma samples and CT scans collected at baseline and following induction systemic therapy from 101 patients with unresectable, liver-limited CRC enrolled in the phase-III CAIRO5 trial (NCT02162563), treated with FOLFOX/FOLFIRI plus bevacizumab. Total tumour volume (TTV) was centrally quantified via semi-automated segmentation of liver metastases. ctDNA was measured using the DELFI-TF score. Associations with overall survival (OS) and early recurrence were evaluated using multivariable Cox regression models.

**Findings:**

At baseline, TTV (median = 139 mL, IQR = 23–497 mL) strongly correlated with DELFI-TF (median = 0.29, IQR = 0.13–0.41; Spearman's ρ = 0.70). DELFI-TF showed a more pronounced reduction than TTV on-treatment (−97.6% vs −49.9%). Baseline levels and on-treatment changes of DELFI-TF (*P* = 0.001; *P* = 0.012) and TTV (*P* = 0.002; *P* = 0.002) were independently associated with OS in the multivariable model; their combination improved prognostic performance (Uno's C-statistic 0.78 vs 0.73; *P* = 0.036). Baseline (*P* = 0.016) and on-treatment DELFI-TF (*P* = 0.001) also predicted early recurrence after local therapy.

**Interpretation:**

Following further validation, integrating cfDNA fragmentome-based testing with radiological tumour volume may provide complementary and clinically meaningful insights for prognostication and treatment response in patients with unresectable CRLM. This exploratory study supports a multimodal biomarker approach to guide personalised treatment strategies.

**Funding:**

10.13039/501100001659German Research Foundation (DFG, 513004649), Heidelberg Medical Faculty, 10.13039/501100004622Dutch Cancer Society/10.13039/501100004622KWF Kankerbestrijding (10438), PPP Allowance via Health ∼ Holland (LSHM22027), Dr. Miriam and Sheldon G. Adelson Medical Research Foundation, Stand Up To Cancer (SU2C)in-Time Lung Cancer Interception Dream Team Grant, SU2C–Dutch Cancer Society International Translational Cancer Research Dream Team Grant (SU2C-AACR-DT1415), 10.13039/100015616Gray Foundation, 10.13039/100004436Commonwealth Foundation, 10.13039/100019683Cole Foundation, Delfi Diagnostics (research grant), US 10.13039/100000002National Institutes of Health (CA121113, CA233259, CA271896).

**Research in context**

**Evidence before this study**

Circulating tumour DNA (ctDNA) and radiological imaging are increasingly studied as tools for response monitoring and prognostication in metastatic colorectal cancer (CRC). Several studies have shown that ctDNA levels and radiological parameters may independently predict survival outcomes. However, most of these studies evaluated ctDNA and imaging biomarkers separately, were limited to resectable disease or focused on mutation-based ctDNA approaches that require tumour tissue. Prognostic biomarkers specifically for patients with initially unresectable colorectal cancer liver metastases (CRLM) are rare, and there is a lack of integrated analyses combining molecular and radiological tumour burden features in this setting.

**Added value of this study**

This study evaluates the combined prognostic value of radiological tumour burden and a non-tumour informed, fragmentome-based circulating tumour DNA (ctDNA) detection technique in patients with initially unresectable CRLM, using data from the randomised multicenter phase III CAIRO5 trial. We integrated total tumour volume (TTV) measured from CT imaging and the DELFI-TF score derived from plasma cell-free DNA to assess treatment response and survival outcomes. DELFI-TF is a tumour tissue- and mutation-independent ctDNA detection technique, thereby offering a broadly applicable assay for treatment response evaluation. Both TTV and DELFI-TF were independently associated with overall survival and early recurrence. Importantly, their combination significantly improves risk stratification beyond standard clinicopathological features. We also show that ctDNA levels decline more sharply than radiological tumour volume during treatment, highlighting its potential as a dynamic and sensitive marker of response in this clinical setting. The observed interaction between molecular and radiological tumour burden suggests that patients with low-volume disease may still carry poor prognosis if ctDNA levels are elevated, offering clinically relevant insights for treatment planning. These findings support a more integrated, biomarker-informed approach to managing unresectable CRLM that does not rely on prior tumour tissue, provided that such biomarker assessments undergo robust clinical validation to ensure accuracy, reproducibility, and clinical utility.

**Implications of all the available evidence**

Our findings provide a rationale for incorporating both ctDNA and TTV into clinical workflows for CRLM. Following further validation, this dual-biomarker strategy could potentially enhance personalised treatment decisions, identify patients who may benefit from local therapy despite modest radiological response, and avoid potentially ineffective treatment in those with persistent high ctDNA. These findings pave the way for future trials aimed at stratifying patients with CRLM for neoadjuvant vs upfront surgeries, and for incorporating ctDNA dynamics as a meaningful endpoint in studies evaluating treatment response.

## Introduction

Liver metastases are commonly diagnosed in patients with colorectal cancer (CRC) and are the leading cause of death in these patients.^1^ Treatment decisions for patients with initially unresectable colorectal cancer liver metastases (CRLM) are currently guided by technical resectability, which includes assessment of future liver remnant, and response to induction systemic therapy.^2^ However, due to the limited number of studies and the lack of a standardised definition for surgical resectability, reaching a consensus on the best diagnostic and therapeutic strategies remains challenging.^2^, ^3^, ^4^, ^5^ Recurrence rates remain high, with over 70% of cases recurring within one year post-surgery.^6^ While molecular characteristics of CRC are increasingly recognised as essential for predicting patient outcomes, the incorporation of these findings in clinical decision-making has been limited.^5^^,^^7^, ^8^, ^9^, ^10^, ^11^ The intricacy of CRLM management lies in balancing the technical aspects of resectability with the biological complexity of metastatic disease.^7^ Most critically, biomarkers to reliably predict treatment response, long-term survival, or recurrence risk in these patients are scarce.^5^ This underscores the need for better diagnostic and therapeutic strategies to guide personalised treatment-decision making for patients with CRLM.

Recently, baseline total tumour volume (TTV) and its change during systemic treatment in CT scans of patients with unresectable CRLM were shown to be highly prognostic and they both outperformed conventional clinicopathological variables including treatment response assessment using RECIST.^12^ Despite promising findings of TTV, the limitations of radiological evaluation persist, such as the need for significant macroscopic changes to detect variation, lack of resolution to assess microscopic disease burden and inter-reader variability of non-automated measurements.^13^ The analysis of circulating tumour DNA (ctDNA) introduces a novel and promising biomarker in oncology, capturing tumour-related genetic and epigenetic alterations in liquid biopsies.^14^^,^^15^ An important challenge is to avoid false positive results due to clonal haematopoietic variants.^16^ Most commonly used technologies for measuring ctDNA rely on detecting specific mutations, typically by sequencing known cancer genes or tracking previously identified tumour mutations using techniques such as droplet digital PCR (ddPCR) or targeted next-generation sequencing (NGS). While effective, these mutation-based approaches require prior knowledge of the tumour's genomic profile and necessitate access to tissue biopsies. This introduces logistical challenges, delays, and additional costs.

In contrast, the DELFI approach (‘DNA evaluation of fragments for early Interception’) leverages a fundamental biological difference between tumour-derived and normal cell-free DNA (cfDNA): fragmentation patterns.^17^, ^18^, ^19^, ^20^, ^21^, ^22^ Tumour-derived cfDNA tends to exhibit shorter, more irregular fragment lengths and specific genome-wide fragmentation profiles, known as the cfDNA fragmentome.^23^ By applying machine learning on low-coverage whole-genome sequencing data of plasma cfDNA, the DELFI method can quantify the tumour-derived fraction of cfDNA, yielding the DELFI tumour fraction (DELFI-TF) score.^17^^,^^18^ Importantly, DELFI-TF does not rely on prior knowledge of tumour mutations and is both tumour tissue- and tumour type-independent, making it a broadly applicable ctDNA assay across cancer types. It eliminates the need for tissue biopsies and enables non-invasive, real-time assessment of tumour burden and treatment response, offering a scalable alternative to mutation-based ctDNA assays. While the DELFI approach has so far been mostly investigated in the setting of early detection,^15^^,^^24^^,^^25^ its clinical application for treatment response monitoring is emerging.^18^^,^^26^^,^^27^

Although ctDNA shedding is associated with tumour volume, the levels of ctDNA are also influenced by biological factors like histological subtype and location.^28^, ^29^, ^30^ Therefore, the DELFI-TF score, which measures tumour burden at the molecular level, offers potential as a complementary tool to radiological imaging for predicting treatment response and survival outcomes.^13^^,^^31^ Previous studies have demonstrated that radiological assessment and ctDNA are independently associated with prognosis and treatment response monitoring in metastatic CRC, although these associations were evaluated in separate studies rather than concurrently. Therefore, it remains to be established whether they provide complementary insights in addressing these clinical objectives.^12^^,^^13^^,^^32^

We hypothesised that integrating radiological TTV with ctDNA testing using DELFI-TF may enhance the prediction of clinical outcomes in patients with CRLM. The aim of this study was to examine the relationship between TTV and DELFI-TF scores and to evaluate their individual and combined value for predicting the outcome and treatment response of patients with unresectable CRLM.

## Methods

### Study design and study population

In this translational study, we included patients who participated in the phase III randomised CAIRO5 trial (NCT02162563),^33^, ^34^, ^35^ and had at least one blood draw before and after treatment initiation,^18^ and quantified TTV before and after treatment initiation.^12^ Blinding was not applicable to this study, as all analyses were based on previously collected clinical and imaging data from the prospective CAIRO5 trial. No formal power analysis was conducted, as this was an exploratory translational study utilising available samples from the CAIRO5 trial.

The CAIRO5 trial investigated the optimal first-line systemic therapy for patients with histologically proven CRC with isolated, chemo-naive, and initially unresectable CRLM. At inclusion, all patients were deemed unresectable, meaning an R0 resection could not be achieved in a single surgical procedure. Following treatment, patients were assessed every two months by an expert panel comprising liver surgeons and abdominal radiologists to evaluate the feasibility of local treatment for CRLM in line with current clinical practice.^36^ Clinical follow-up adhered to standard care protocols, including clinical reviews every three months, as well as CT imaging and serum carcinoembryonic antigen (CEA) measurements every six months. For patients whose CRLM remained unresectable, chemotherapy was continued without the targeted agent. Continuous evaluation was carried out through serum CEA levels and CT imaging every two months until disease progression. Follow-up was recorded until February 7, 2024. All patients who were included in this study were treated between March 2015 and November 2020 with doublet chemotherapy (folinic acid, fluorouracil, and oxaliplatin (FOLFOX) or folinic acid, fluorouracil, and irinotecan (FOLFIRI)) and bevacizumab.

Baseline demographic and clinical characteristics, including age, sex as biological variables, WHO performance status, number of metastases, serum CEA levels, and *KRAS/BRAF*^*V600E*^ mutation status, were collected for all patients at the time of inclusion.

As this was a retrospective post-hoc analysis of clinical trial data, there was no direct involvement of patients or the public in the study design, conduct, or dissemination.

### Ethics

The study was approved by the Institutional Review Board (CFMPB 387). The collection of the blood samples and CT scans was part of the CAIRO 5 study, which was approved by the medical ethical committee of the Amsterdam University Medical Centre, Amsterdam. All patients provided written informed consent for participation and blood collection for translational research.

### Total tumour volume quantification

In pre-treatment and the first post-treatment portovenous CT scans, all CRLM were segmented with semi-automatic software in the Tumour Tracking Modality of IntelliSpace Portal 9.0® (Philips, Best, The Netherlands) by one trained member of the research team (J.M.Z., N.W., R.K.). All segmentations were verified and, if needed, adjusted by one experienced abdominal radiologist (J.H.v.W., J.v.d.B., S.M., I.N.). TTV was calculated in the SAS analytical platform® (SAS Viya 3.5, SAS Institute Inc.) using the quantifyBioMedImages action. This action calculates TTV directly out of the tumour segmentation from all CRLM, based on the voxel size and the number of voxels included in the segmentation.^37^ TTV was assessed at baseline prior to systemic induction therapy and at the first post-treatment scan. To determine TTV response to systemic therapy, the absolute difference in pre-treatment TTV and first post-treatment TTV was calculated in millilitres.

### ctDNA detection methodology

The DELFI-TF score is a tumour-type-independent and mutation-independent, cfDNA fragmentome-based approach to determine the level of ctDNA in patients with cancer. It utilises low-coverage whole genome sequencing (∼6×) of cfDNA from plasma and machine learning analyses of genomic and fragmentomic features, as described previously.^17^^,^^18^

### Blood collection and cfDNA extraction and sequencing

Collection of liquid biopsy samples was performed at the medical centre of inclusion prior to study treatment (baseline) and longitudinally every three months during follow-up until disease progression or treatment completion. For this study, blood samples collected at baseline and after induction systemic therapy were used. Details about the sample and library preparation, as well as the cfDNA sequencing have been described previously.^18^ Briefly, blood samples were collected in 10 mL cell-free DNA BCT® tubes and centrally processed at the Netherlands Cancer Institute. Plasma was isolated using a two-step centrifugation process (1700×*g* for 10 min, followed by 20,000×*g* for 10 min) and stored at −80 °C. cfDNA was extracted using the QIAsymphony robot and quantified with the Qubit High-Sensitivity assay (Thermo Fisher Scientific, Waltham, MA; cat #Q33231). Aliquots of 15 ng cfDNA were used to prepare NGS libraries using the NEBNext DNA Library Prep kit (New England Biolabs; Ipswich, MA, USA, cat #E7645) and purified with AMPure XP beads (Beckman Coulter; Brea, CA, USA, cat #A6388). Libraries were amplified using Phusion HotStart Polymerase (ThermoFisher; Waltham, MA, USA, cat #F549S) and assessed for quality using the 2100 Bioanalyzer (Agilent Technologies; Santa Clara, CA, USA) or TapeStation 4200 (Agilent Technologies; Santa Clara, CA, USA). Sequencing was performed on an Illumina NovaSeq 6000 platform with a target depth of approximately 6× coverage.^18^ Sequenced reads were aligned to the reference genome (hg19) using Bowtie 2 (version 2.4.2, RRID:SCR_005476) and converted to BED format with Samtools (version 1.31, RRID:SCR_002105) and Bedtools (version 2.26.0, RRID:SCR_006646 RRID:SCR_002105). cfDNA-derived features were integrated into a random forest model to predict the mutant allele frequency (MAF) representing the DELFI-TF score, based on short-to-long fragment ratios, chromosomal arm changes, and overall fragment-length distribution.^18^ The DELFI-TF scores used in the present study were generated in the original study by van 't Erve et al.^18^

### Outcomes

Overall survival (OS) was defined as time from randomisation to death from any cause. Recurrence-free survival (RFS) was calculated from date of first liver procedure to date of progression. In case of a two-stage resection, RFS was calculated from last liver procedure. Complete resection was defined when all CRLM were treated with an R0-R1 resection or ablation of all CRLM, or both. Early recurrence was defined as any recurrence within 6 months after surgery. None of the patients died or were censored before early recurrence, i.e., there were no competing risks for RFS.

### Aims

The primary aim of this study was to investigate the independent prognostic value of total tumour volume and ctDNA (quantified using DELFI-TF) for overall survival. Secondary aims were to assess (i) the correlation between TTV and DELFI-TF prior to and during systemic treatment, and (ii) the prognostic value of TTV and DELFI-TF for early recurrence (<6 months after local treatment).

### Statistics

Continuous and categorical baseline characteristics are presented as median (interquartile range [IQR]) or as frequencies and percentages. The Mann–Whitney U test was used to compare continuous variables (c.q., TTV and DELFI-TF scores) between patients with and without early recurrence. OS curves were generated using the standard Kaplan–Meier method. Median follow-up was estimated using the reverse Kaplan–Meier method,^38^ which accounts for censoring and calculates follow-up based on the time until censoring.

Correlation between TTV and DELFI-TF, both at baseline and after systemic treatment, was quantified using Spearman's ρ. Nonparametric bootstrapping based on 1000 bootstrap samples was used to derive bias-corrected and accelerated 95% confidence intervals. The relative change of DELFI-TF after initiation of treatment was defined as ratio between ctDNA on-treatment and baseline ctDNA.^39^ An interaction analysis was performed using bootstrapping to assess whether the correlation between TTV and DELFI-TF parameters was similar between patients with and without the primary tumour present.

To assess the prognostic value of TTV and DELFI-TF for OS we used multivariable Cox regression modelling, with the following covariates: (i) baseline TTV, baseline DELFI-TF, change in TTV following systemic treatment, and change in DELFI-TF following systemic treatment; (ii) balancing variables used in the minimisation procedure for the CAIRO5 trial, i.e., resectability at baseline, serum lactate dehydrogenase concentration, and *KRAS/BRAF*^*V600E*^ mutation status; and (iii) additional known prognostic factors in patients with CRLM, i.e., age at diagnosis, number of metastases, baseline CEA levels, metachronous/synchronous metastases, WHO performance score and sex.^12^ To model the joint association of TTV and DELFI-TF in this multivariable model, we also included (i) an interaction term between baseline TTV and baseline DELFI-TF, and (ii) an interaction term between change in TTV and change in DELFI-TF following systemic therapy.

To assess whether the prognostic value of baseline and change in TTV and DELFI-TF was modified by the delay in the measurement of TTV and DELFI-TF, an interaction analysis was performed using Cox regression with multiplicative interaction terms between the delay variable (i.e., the difference in days between the TTV and DELFI-TF measurement) and the TTV and DELFI-TF variables.

All continuous variables were modelled using restricted cubic splines to allow for nonlinear covariate–outcome associations. The proportional hazards assumption was assessed using the Grambsch–Therneau test and visual inspection of Schoenfeld residuals. The prognostic value of all included covariates and interactions was determined using likelihood ratio tests. For both recurrence and OS, we assessed whether relative or absolute change in TTV should be used for subsequent analysis using likelihood ratio tests.

To visualise the joint association of baseline TTV and DELFI-TF with OS, flexible parametric survival models were used, adjusted for the same variables as in the Cox regression. Regression standardisation was used to derive adjusted 3-year OS probabilities. Confidence intervals were obtained using the delta method.

A *P*-value lower than 0.05 was considered to indicate statistical significance. All statistical analyses were performed using R, version 4.5.0 (R Foundation for Statistical Computing, Vienna, Austria), and Stata, version 17 (StataCorp), using the following packages: tidyverse, rms, and flexsurv.

### Role of funders

The funding source had no role in the study design, data collection, data analysis, data interpretation, writing of the report, or the decision to submit the paper for publication.

## Results

### Patient and sample characteristics

In total, 521 patients were included in the modified intention-to-treat analysis of the CAIRO5 trial, of whom 101 (19%) were eligible for the present study (Supplementary Fig. S1). Patient characteristics are summarised in Table 1. Among the included patients, 45 patients (45%) had a *KRAS* mutation and 19 patients (19%) a right sided primary tumour. In total, 97 patients (96%) received FOLFOX plus bevacizumab and 4 patients (4%) FOLFIRI plus bevacizumab. After systemic induction therapy, local therapy (resection with or without local ablative treatments) was considered suitable in 52 patients (51%). In 46 of these patients (90.2%), complete local treatment was achieved, and they were included in the subgroup analysis for the association with early recurrence.

**Table 1:** Overview of the clinicopathological characteristics of the patient cohort.

Characteristic	N = 101; median (IQR); N (%)
**Age (years)**	59 (53, 66)
**Sex**	
Female	39 (39%)
Male	62 (61%)
**WHO performance status**	
0	59 (59%)
I	41 (41%)
**Sidedness of the primary tumour**	
Left	82 (81%)
Right	19 (19%)
**KRAS mutation**	45 (45%)
**BRAF V600E mutation**	7 (6.9%)
**Primary resected at baseline**	31 (31%)
**Time of metastasis**	
Metachronous	8 (8%)
Synchronous	93 (92%)
**Median number of liver metastases**	17 (8, 33)
**Chemotherapy type**	
FOLFIRI-Bevacizumab	4 (4.0%)
FOLFOX-Bevacizumab	97 (96%)
**Number of induction cycles**	8 (6, 12)
**Considered suitable for local therapy after induction**	52 (51%)
**Type of local therapy**	
No local treatment	49 (49%)
Surgery + Local ablative treatment/Radiotherapy	27 (27%)
Surgery only	25 (25%)
**Resection strategy**	
1-stage	38 (38%)
2-stages	14 (14%)
N/A	49 (49%)
**Complete local therapy**	46 (46%)
**Adjuvant therapy**	
No	28 (28%)
Yes	24 (24%)
N/A	49 (49%)
**Total Tumour volume (ml)**	139 (23, 497)
**DELFI Tumour fraction score**	0.29 (0.13, 0.41)

After a median follow-up of 64 months (IQR, 61–70), 82 patients (81%) had died with a median overall survival (OS) of 30 months (25–34 months), Supplementary Fig. S2. Of the 46 patients that were included in the subgroup analysis of the resected patients, 19 patients (41%) developed early recurrence. None of these patients died before 6 months after surgery or were lost to follow-up.

There was a median time between baseline CT scan and baseline blood draw of 22 days (IQR, 14–32 days) and a median time between the CT scan and blood draw on-treatment of 6 days (IQR, 2–9 days).

### Total tumour volume characteristics

**Fig. 1:**
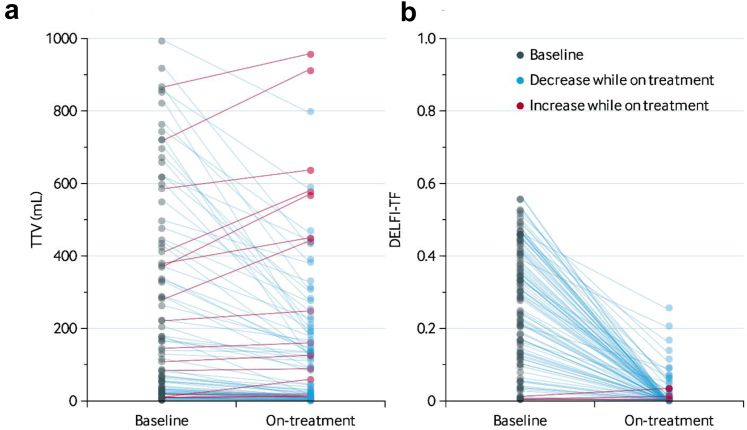
**TTV and DELFI-TF at baseline and on systemic treatment.** (a) Line plot showing total tumour volume (TTV) values on the y-axis and the two timepoints, baseline and on systemic treatment, on the x-axis. Data are presented for patients with TTV values below 1000 mL. (b) Line plot showing DELFI-TF values on the y-axis and the two timepoints, baseline and on systemic treatment, on the x-axis. Patients with an increase are shown in red and patients with a decrease are shown in blue.

The median TTV at baseline was 139 mL (IQR, 23–497 mL). At first CT scan after four cycles of induction systemic therapy, median TTV was 92 mL (IQR, 11–276 mL). The median change in TTV was −27 mL (IQR, −171 to −4 mL) and the median relative change −49.9% (−63.8 to −27.7). An overview of the changes between TTV at baseline and on-treatment are shown in Fig. 1a. A detailed overview of the individual TTV values at baseline and on treatment are provided in Supplementary Table S1.

### DELFI-TF characteristics

The median DELFI-TF score at baseline was 0.29 (IQR, 0.13–0.41). At first CT scan after induction systemic therapy, median DELFI-TF score was 0.005 (IQR, 0.002–0.011). The median change in DELFI-TF-score was −0.24 (−0.11 to −0.37) and the median relative change −97.6% (−99.0 to −90.9). An overview of the changes between DELFI-TF score at baseline and on-treatment are shown in Fig. 1b and Supplementary Table S1. These data show that the dynamic changes in DELFI-TF upon treatment are more profound than the dynamic changes in TTV.

### Correlation between TTV and DELFI-TF

**Fig. 2:**
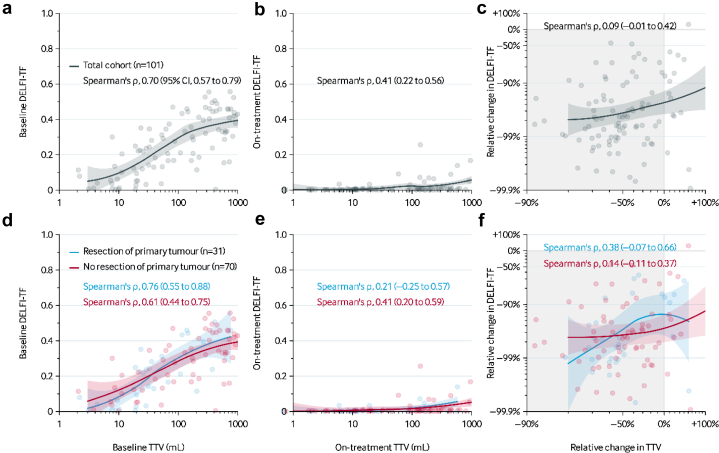
**Correlation between TTV and DELFI-TF.** (a) Correlation between TTV and DELFI-TF at baseline. (b) Correlation between TTV and DELFI-TF on-treatment. (c) Correlation between the relative change in TTV and relative change in DELFI-TF on-treatment. (d) Correlation between TTV and DELFI-TF at baseline stratified by whether the primary tumour was present at baseline (n = 70) or not (n = 31). (e) Correlation between TTV and DELFI-TF on-treatment, stratified by whether the primary tumour was present at baseline. (f) Correlation between the relative change in TTV and relative change in DELFI-TF on-treatment when stratified by whether the primary tumour was present at baseline. Trend lines are based on nonparametric LOESS regression. Baseline TTV, relative change in TTV, and relative change in DELFI-TF are shown on a log-transformed scale. The light-grey region in panels c and f indicates a decrease in TTV and DELFI-TF. Abbreviations: CI, confidence interval; DELFI-TF, DELFI tumour fraction; TTV, total tumour volume.

To better understand the relationship between radiological and molecular assessments of tumour burden, we analysed the correlation between baseline TTV and DELFI-TF, as well as their changes during systemic treatment. At baseline, there was a moderate positive correlation between TTVs and DELFI-TF scores (Spearman's ρ, 0.70 [95% CI, 0.57–0.79]; Fig. 2a). In contrast, on-treatment, the correlation dropped (Spearman's ρ, 0.41 [95% CI, 0.22–0.56]; Fig. 2b). There was no evidence for a correlation between the change in TTV and the change in DELFI-TF score between these two timepoints (Spearman's ρ, 0.09 [–0.01 to 0.42]; Fig. 2c).

At the baseline measurements, 70 patients (69%) had their primary tumour still present, which was not included in the TTV measurements. To account for this, we performed separate correlation analyses between TTV and DELFI-TF for patients with and without a primary tumour in situ at baseline. The results are presented in Fig. 2d–f.

The correlation between TTV and DELFI-TF was similar between patients with and without resection of the primary tumour, both at baseline (*P* = 0.19; bootstrap test) and while on-treatment (*P* = 0.39; bootstrap test).

### Prognostic value of TTV and DELFI-TF for overall survival

**Fig. 3:**
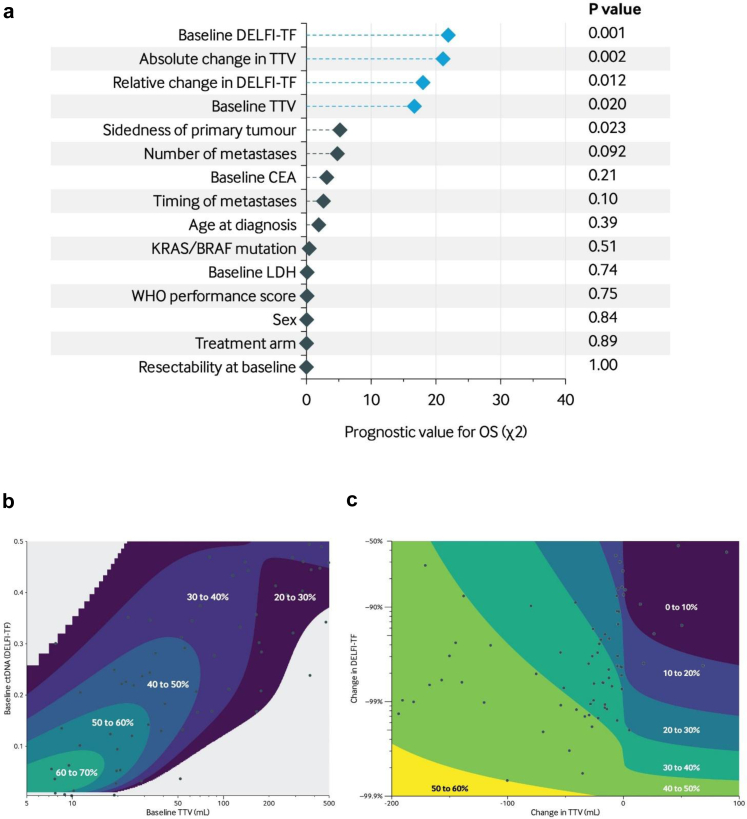
**Prognostic value of predictors for OS and interaction between TTV and DELFI-TF.** (a) Multivariable analysis with the OS as outcome variable. The model contains all variables listed in the plot as well as the (I) interaction between baseline TTV and baseline DELFI-TF (*P*_interaction_ = 0.002); (II) interaction between absolute change in TTV and relative change in DELFI-TF (*P*_interaction_ = 0.008). The interaction chi-squares are not displayed, as they are already accounted for in the chi-squared values of the respective TTV and DELFI-TF variables, making their inclusion redundant. The x-axis shows the prognostic value for OS, while the y-axis lists the variables included in the multivariable model. Variables associated with DELFI-TF or TTV are blue, other clinicopathological characteristics are teal. (b) Association of baseline TTV (x-axis) and baseline DELFI-TF score (y-axis) with 3-year OS probability (z-axis). Regions with a low number of data points are shown in grey to avoid extrapolation. (c) Association of absolute change in TTV (x-axis) and relative change in DELFI-TF score (y-axis) on-treatment with 3-year OS probability (z-axis).

We used Cox regression to assess the added value of TTV and DELFI-TF through adjustment for clinicopathological variables, and to investigate whether DELFI-TF modified the association between TTV and OS using interaction terms (Fig. 3). Baseline TTV (*P* = 0.020; likelihood ratio test), absolute change in TTV (*P* = 0.002; likelihood ratio test), baseline DELFI-TF score (*P* = 0.001; likelihood ratio test) and relative change in DELFI-TF score (*P* = 0.012; likelihood ratio test) emerged as the strongest predictors for OS (Fig. 3a). Among the variables analysed, the prognostic value of TTV and DELFI-TF-related metrics was more pronounced than tumour sidedness (*P* = 0.023; likelihood ratio test), while other clinicopathological features, including baseline CEA levels (*P* = 0.210; likelihood ratio test), were not significantly prognostic for OS. By combining DELFI-TF with TTV in this model, we observed a substantial improvement in prognostic performance (Uno's C-statistic, 0.78 vs 0.73; difference, 0.05 [0.01–0.10]; *P* = 0.036; bootstrap test).

### Interaction between TTV and DELFI-TF

We investigated the putative interaction between TTV and DELFI-TF using the same Cox regression model and observed a ctDNA-dependent association between baseline TTV and OS (*P*_interaction_ = 0.002; likelihood ratio test) and a similar interaction effect in the absolute change in TTV and relative change of DELFI-TF score on systemic treatment (*P*_interaction_ = 0.008; likelihood ratio test).

First, we investigated the interaction between baseline TTV and baseline DELFI-TF and their combined influence on OS using flexible parametric survival models (Fig. 3b). Notably, 3-year OS was similar between patients with low TTV and high ctDNA compared to patients with high TTV and low ctDNA. For instance, 3-year OS was 42% at a baseline TTV of 10 mL and a DELFI-TF score of 0.2 *vs* a 3-year OS of 42% at a baseline TTV of 40 mL and a DELFI-TF of 0.1 (difference, 0%; [95% CI, −27 to +28%]). In addition, we investigated the interaction effects in the absolute change in TTV and relative change in DELFI-TF on systemic treatment. Notably, 3-year OS was similar for patients with a strong decrease in TTV and a modest decrease in DELFI-TF compared to patients with a modest decrease in TTV and strong decrease in DELFI-TF (Fig. 3c).

There was no evidence that the interaction between TTV and DELFI-TF was modified by the time delay between their respective measurements, neither at baseline (*P* = 0.78; likelihood ratio test) nor at the on-treatment timepoint (*P* = 0.48; likelihood ratio test).

### Prognostic value of TTV and DELFI-TF for early recurrence

**Fig. 4:**
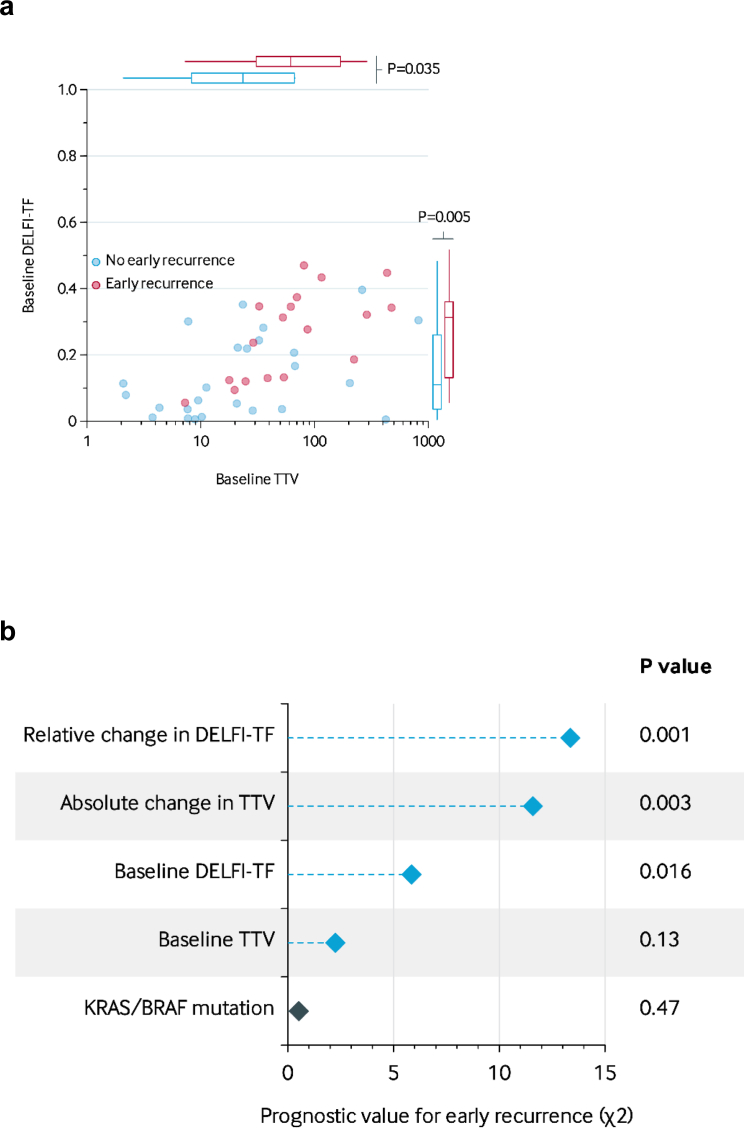
**Prognostic value of predictors for early recurrence.** (a) Correlation between baseline TTV (x-axis) and baseline DELFI-TF score (y-axis) on the development of an early recurrence. (b) Prognostic value of predictors for early recurrence. Multivariable analysis with the early recurrence as outcome variable. The x-axis shows the prognostic value for early recurrence, while the y-axis lists the variables included in the multivariable model. Variables associated with DELFI-TF or TTV are blue, other clinicopathological characteristics are teal.

We further analysed the association of TTV and DELFI-TF for early recurrence in the 46 patients who underwent complete local treatment. Patients with early recurrence (*N* = 19) showed higher baseline DELFI-TF scores (median 0.31 [IQR, 0.13–0.36]) and baseline TTV (median 62 mL [31–169 mL]) than patients with no early-recurrences (*N* = 27; median DELFI-TF score 0.11 [0.04–0.26], *P* = 0.005; median TTV 24 mL [8–67 mL], *P* = 0.035; Mann–Whitney U test; Fig. 4a). Next, we integrated the TTV and DELFI-TF metrics into a multivariable model for early recurrence as outcome. Due to the small sample size, we adjusted only for the *KRAS/BRAF*^*V600E*^ mutation status. The baseline DELFI-TF (*P* = 0.016; likelihood ratio test) and the relative change in DELFI-TF (*P* = 0.001; likelihood ratio test) as well as the absolute change in TTV (*P* = 0.003; likelihood ratio test) were significantly prognostic for the occurrence of an early recurrence (Fig. 4b).

## Discussion

TTV and DELFI-TF are both indicative for tumour burden. However, they measure distinct biological features representing tumour volume and relative amounts of (dying) cancer cells, respectively, which could have important clinical implications.^31^ This study shows that TTV and DELFI-TF scores independently outperformed conventional clinical prognostic factors in predicting OS in patients with initially unresectable CRLM, as well as early recurrence in patients undergoing local treatment after systemic therapy. DELFI-TF scores and TTV at baseline, along with their changes during systemic treatment, were independently associated with OS. Notably, combining tumour-independent and mutation-independent DELFI-TF ctDNA testing and volumetric measurements in one overall survival model significantly enhanced the prognostic performance. The increase in the C-statistic is substantial, given the challenge of achieving high C-statistics in survival models where time-dependent outcomes and censoring often deflate the concordance calculation.^40^

During systemic treatment, both TTV and DELFI-TF scores demonstrated significant reductions compared to baseline levels, though the extent and patterns of these changes differed. ctDNA levels decreased proportionally more than TTV, highlighting DELFI-TF's potential as a dynamic marker of treatment response. While TTV reflects radiologically visible tumour masses composed of cancer cells and the tumour microenvironment with non-cancer cells and extracellular matrix, DELFI-TF may better represent the relative amount of viable cancer cells. This may explain why the molecular reduction in DELFI-TF upon systemic therapy is more profound than the radiological reduction in TTV. However, the underlying biology of ctDNA shedding in general remains poorly understood and warrants further mechanistic studies.

The observed prognostic impact of baseline TTV on OS being modulated by DELFI-TF levels highlights the nuanced relationship between radiological and molecular features. For instance, patients with low TTV but high ctDNA exhibited similar survival outcomes to those with higher TTV but lower ctDNA. This highlights the importance of integrating ctDNA into clinical assessment, as even patients with a seemingly low radiological tumour burden might still have a poor prognosis if their ctDNA levels are elevated. Similarly, during systemic treatment, the finding that a high reduction in ctDNA corresponded to better outcomes, even when accompanied by only modest reductions in TTV, underscores the dynamic sensitivity of liquid biopsies in detecting treatment responses at a molecular level that is not readily captured by radiological methods.

We previously demonstrated that RECIST 1.1 is not prognostic in CRLM.^12^^,^^37^ Therefore, we used the TTV as a corresponding radiological measurement of tumour burden. The complementary insights provided by TTV and ctDNA are particularly relevant for guiding treatment decisions in complex cases like initially unresectable CRLM, where biomarkers to inform prognosis and treatment response are currently lacking. By considering both biomarkers together, clinicians can achieve a more holistic understanding of disease burden and its evolution during treatment. For example, a patient with a significant decrease in ctDNA but modest radiological changes could be identified as a candidate for more aggressive local therapies or monitored closely for potential recurrence. Conversely, patients with stable radiological assessments but persistently high ctDNA might warrant alternative therapeutic approaches or intensified systemic treatments, reflecting the aggressive underlying disease suggested by the ctDNA profile.

Several studies have demonstrated prognostic value of mutation-based ctDNA-testing in patients with CRLM, primarily focussing on postoperative ctDNA in case of resectable liver metastases.^41^, ^42^, ^43^, ^44^ Importantly, the DELFI-TF fragmentome-based ctDNA detection method used in the current study is mutation-independent and tumour-type-independent, eliminating the need for tumour tissue profiling to obtain a view of the patient's tumour landscape. These advantages allow for the application of a single ctDNA assay across the entire patient cohort, offering a practical and scalable advantage for clinical management of CRLM.^17^^,^^18^

Most studies on radiological imaging and ctDNA have focused on comparing the two diagnostic approaches to assess which is more reliable in detecting disease progression.^16^^,^^32^^,^^45^^,^^46^ In the present study all patients had liver-limited metastases, which tend to shed ctDNA well, and we observed a high correlation between ctDNA and radiological tumour burden. However, results have been inconsistent across various cancer types. Some studies have shown a modest positive correlation between plasma ctDNA levels and total tumour burden, with stronger associations observed during disease progression, while others have found a strong correlation at baseline, but not during progression.^47^, ^48^, ^49^, ^50^ Several studies have attempted to integrate these two features, demonstrating improved risk stratification and complementary utility for predicting OS, treatment response, and recurrence risk.^51^, ^52^, ^53^ However, their integration in metastatic CRC remains limited, and none have focused specifically on cfDNA fragmentomics-based metrics in conjunction with volumetric radiological tumour burden in liver-limited disease. The present study combines the DELFI-based tumour fraction with TTV in this specific clinical context, demonstrating that both biomarkers, while initially correlated, show differential dynamics during treatment. This highlights the potential biological complementarity of these metrics and supports their combined use to improve response evaluation and individual risk stratification in metastatic CRC.

This study has several limitations. The timing of blood draws for ctDNA detection and CT scans was not always aligned, with a median difference of 22 days at baseline (IQR, 14–32 days) and 6 days (2–9 days) during systemic treatment. Although such misalignment may have influenced the results and the correlation between ctDNA levels and radiological assessments, the interaction analyses found no evidence that this delay in timing substantially impacted our results. In addition, the primary tumour was not included in TTV measurements, and the potential impact of its presence vs absence warrants further investigation.

Importantly, we were unable to conduct a formal power calculation in advance because the analysis was based on all available plasma and imaging data from a prospectively enrolled trial, which may in turn affect the generalisability of the findings. The modest sample size, particularly within the resected subgroup, also restricted the scope of analyses. Early recurrence with non-salvageable local therapy options would be an even more critical outcome measure than solely early recurrence,^34^ but the sample size was not sufficient for such an analysis (data not shown). The limited number of events (n = 19) constrained the multivariable analysis to a single biologically relevant covariate (KRAS/BRAF status), as inclusion of additional variables would risk sparse data bias. While adjuvant therapy may influence recurrence risk, it represents a post-resection factor and thus is not suitable for inclusion in baseline-adjusted prognostic models. Subgroup analysis would be more suitable in this context, but was again hampered by small numbers.

Ultimately, validation of these findings in larger, independent cohorts is needed to confirm the observed associations and enable more comprehensive multivariable analyses. However, obtaining such a clinically well-defined, homogeneous patient cohort with high quality, centrally assessed radiological imaging, paired plasma-based biomarker data, along with long term follow-up in patients with initially unresectable, liver limited colorectal cancer is methodologically and logistically challenging and represents a major strength of the present study.

Furthermore, although we observed an increase in C-statistics of 0.05 (0.78 vs 0.73), it is important to keep in mind that high C-statistics are difficult to achieve in survival models^54^ and often change only marginally when new predictors are added, even if these predictors are clinically relevant.^40^^,^^55^^,^^56^ In this light, the observed improvement can be considered meaningful. However, as no external validation cohort was available, these results should be interpreted with caution. Most importantly, a higher C-statistic does not necessarily translate into better clinical decision-making or improved patient outcomes.^57^ The crucial next step is therefore to evaluate whether the combined use of TTV and ctDNA can prospectively improve therapeutic decisions in clinical practice.

Importantly, comprehensive TTV measurements at the time of disease progression were not consistently available in the CAIRO5 trial, which limited our ability to explore correlations during this stage. Moreover, ctDNA dynamics during progression are likely influenced by multiple biological factors, including metastatic site,^30^^,^^58^ treatment resistance,^59^^,^^60^ and tumour heterogeneity,^60^, ^61^, ^62^ which may contribute to the variability observed across prior studies.^47^, ^48^, ^49^, ^50^ Nevertheless, assessing the correlation between TTV and ctDNA at progression remains a key objective and should be prioritised in future studies of CRLM. Lastly, the assessment of TTV was based on manual segmentations by one radiologist using semi-automatic software. Manual segmentation is a labour-intensive and time-consuming task, making TTV assessment less feasible in routine clinical use. To address this, an automatic segmentation model has been developed and validated to facilitate the implementation of TTV assessment in clinical practice.^63^

Beyond the limitations of the present study, the field of ctDNA-based treatment response is also hindered by a lack of standardisation in defining molecular response criteria.^13^^,^^39^^,^^46^ While we demonstrated that the ratio between baseline and on-treatment ctDNA levels had strong prognostic value, the optimal approach for assessing ctDNA response remains a subject of ongoing research.^13^ Different studies in metastatic cancer have applied various metrics, such as ctDNA ratio,^64^^,^^65^ ctDNA clearance,^66^^,^^67^ or specific cut-off points,^16^ to define molecular response, yet there is no consensus on which method best reflects treatment efficacy. This lack of standardisation complicates the interpretation of ctDNA as a tool for treatment response monitoring. Further prospective studies are essential to establish clear and consistent guidelines for defining molecular response based on ctDNA.

This study integrates centrally assessed radiological tumour volume with levels of ctDNA in patients with liver-limited metastatic colorectal cancer, a clinically important subgroup in which prognostic biomarkers for treatment guidance remain elusive. The establishment of robust biomarkers could prevent ineffective systemic treatments and futile surgeries with a significant risk of major complications in patients with CRLM. The prognostic value of baseline DELFI-TF ctDNA in combination with TTV in metastatic CRC could be particularly important for defining oligometastatic disease. While the current definition relies on certain cut-offs, like number of metastatic lesions or patient history,^68^ DELFI-TF and TTV offer a more precise and objective measurement of cancer cell features and tumour burden. This approach can better capture the true extent of disease, as two patients classified as oligometastatic by traditional standards may exhibit vastly different ctDNA levels and tumour volumes, which could directly influence prognosis. Additional research is needed to better understand ctDNA shedding, which is not only indicative for cancer cell burden but also provides information about tumour characteristics.^62^

Prognostic biomarkers for patients with initially unresectable CRLM remain a significant clinical challenge. We here demonstrate that combining radiological tumour burden at CT measured as TTV with cfDNA fragmentome-based ctDNA assessment using the DELFI-TF score provides complementary and clinically relevant insights into risk stratification and response evaluation: while TTV offers a macroscopic view of tumour burden, ctDNA provides a molecular perspective. Nevertheless, certain limitations should be acknowledged, including the incomplete availability of TTV measurements at progression and the biological variability of ctDNA dynamics, which may be influenced by metastatic site, treatment resistance, and tumour heterogeneity. Therefore, prospective validation studies are needed to demonstrate reproducibility and general reliability of our findings, in order to pave the way for a multimodal biomarker strategy to better guide treatment decisions in this patient population.

## Contributors

Conceptualisation: N.C., J.M.Z., N.F.M.K., G.K., R.J.A.F.; Methodology: N.C., J.M.Z., M.A.; Formal analysis: N.C., J.M.Z., M.A. ; Investigation: N.C., J.M.Z., M.A., B.A., K.L., Z.L.S., L.R. , I. v. E., M.J.G.B., A.L.; Resources: J.H., E.G., J.S., I.V., D.v.B., G.A.M., R.J.S., C.J.A.P., R.B.S., N.D., V.E.V., G.K., R.J.A.F.; Data curation: N.C., J.M.Z., N.W., R.K., J.H.v.W., J.v.d.B., I.N., S.M., M.J.G.B., L.M., I.H.; Writing original draft: N.C., J.M.Z., M.A.; Writing, review and editing: all authors; Visualisation: N.C., M.A.; Supervision: E.S., J.S., I.V., J.H., N.F.M.K., G.K., R.J.A.F.; Funding acquisition: N.C., N.W., J.S., I.V., J.H., G.A.M., R.J.S., C.J.A.P., R.B.S., V.E.V., G.K., R.J.A.F.; N.C., J.M.Z., M.A., R.K., R.J.A.F and G. K. have accessed and verified the underlying data. All authors read and approved the final version of the manuscript.

## Data sharing statement

The sequencing data generated in this study have been deposited in the database of the European Genome-Phenome Archive (EGA) and may be obtained at https://egaarchive.org/ under accession codes (EGAS00001006695 [https://ega-archive.org/studies/EGAS00001006695], EGAS00001005340). Requests for access to the EGA deposited data should be addressed to the email dataaccesscommittee.pathology@nki.nl and will be evaluated by the NKI-AvL TGO data access committee and by the NKI-AvL IRB within 6 weeks following the request. Requests should meet GDPR requirements and are pending competitive research efforts. Positive evaluation is followed by establishing a data transfer agreement (∼3–6 months).

## Declaration of interests

G.A.M. is co-founder and board member (CSO) of CRCbioscreen BV, he has a research collaboration with CZ Health Insurances (cash matching to ZonMW grant), he has research collaborations with Sysmex, Sentinel Ch. SpA, Personal Genome Diagnostics (PGDX), Exact Science, DELFi and Hartwig Medical Foundation; these companies provide materials, equipment and/or sample/genomic analyses, and he has several patents pending/issued. He received grants for the study ESCALATION (Dutch Research Council grant with cash or in kind matching by Delfi Diagnostics, RIVM, Palga, Sanquin), COIN (ZonMw grant with cash or in kind matching by CZ Health Insurances, PGDX, IKNL) and WIDE (ZonMw grant with in kind matching by Hartwig Medical Foundation). He has several patents pending related to biomarkers for CRC early detection and related to cfDNA analyses. He has patents pending that are licenced to CRCbioscreen BV (Protein biomarkers (II) for detection of colorectal cancer in stool; Protein biomarkers for detection of colorectal cancer (CRC; Progression markers for colorectal cancer). He is board member and CSO of Health-RI, and in the supervisory board of the IKNL, chair committee in ZonMw, and national co-director of BBMRI-NL and EATRIS-NL.

A.L., R.B.S., and V.E.V. are inventors on patent applications submitted by Johns Hopkins University related to cell-free DNA for cancer detection. A.L., N.C.D., and R.B.S. are founders of DELFI Diagnostics, and R.B.S and AL are consultants for this organisation. L.R., Z.L.S., and K.L are employees, B.A. and A.L. were employees and own DELFI Diagnostics stock. A.L. has pending patents submitted by Johns Hopkins University and royalties from patents licenced through Johns Hopkins University to Delfi Diagnostics. R.B.S. is consultant for Artemyx and owns Artemyx stocks.

V.E.V. is a founder of Delfi Diagnostics, serves on the Board of Directors and as an officer for this organisation, and owns Delfi Diagnostics stock, which is subject to certain restrictions under university policy. Additionally, Johns Hopkins University owns equity in Delfi Diagnostics. V.E.V. divested his equity in Personal Genome Diagnostics (PGDx) to LabCorp in February 2022. V.E.V. is an inventor on patent applications submitted by Johns Hopkins University related to cancer genomic and cell-free DNA analyses that have been licenced to one or more entities, including Delfi Diagnostics, LabCorp, Qiagen, Sysmex, Agios, Genzyme, Esoterix, Ventana and ManaT Bio. Under the terms of these licence agreements, the University and inventors are entitled to fees and royalty distributions. V.E.V. is an advisor to Viron Therapeutics and Epitope. These arrangements have been reviewed and approved by the Johns Hopkins University in accordance with its conflict-of-interest policies.

R.J.A.F reports public private partnership consortia grants in collaboration with Delfi Diagnostics, Natera and with Solvias (Cergentis BV), Labcorp (Personal Genome Diagnostics), MERCK BV, outside the submitted work. In addition, R.J.A.F. has several patents pending. JS is the president of European Society of Gastrointestinal and Abdominal Radiology. DvdB received centrifuge from Delfi Diagnostics.

The remaining authors declare no competing interests.

## Appendix A Supplementary data

Supplementary Figure S2

Supplementary Table S1 Detailed overview of the individual DELFI-TF and TTV values at baseline and on treatment, as well as the individual values for age, sex, arm and presence of primary tumor at baseline.
