# Supplementary figures and images for: Combining cell-free DNA fragmentomes and total tumour volume improves prognostication and tumour response evaluation in patients with colorectal cancer liver metastases
Source: eBioMedicine. 2025 Dec 16;123:106081. doi: 10.1016/j.ebiom.2025.106081 (PMC12768872; doi:10.1016/j.ebiom.2025.106081)

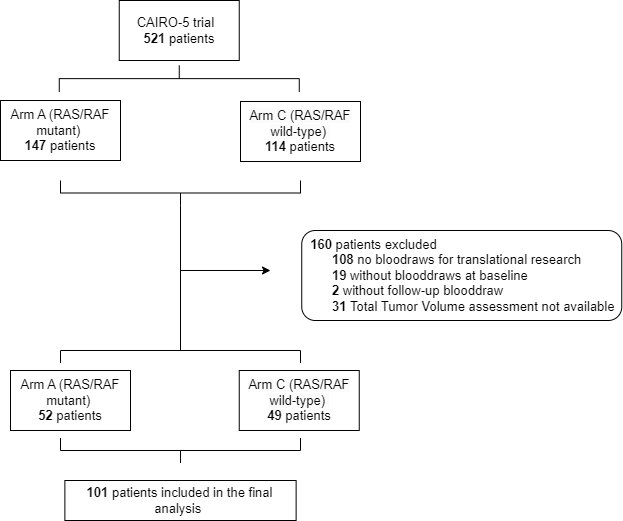


**Suppl. Figure 1.** Flowchart of the study population.

Supplement: Supplementary Figure S1 [file mmc1.docx]
