# Supplementary material for: Combining cell-free DNA fragmentomes and total tumour volume improves prognostication and tumour response evaluation in patients with colorectal cancer liver metastases
Source: eBioMedicine. 2025 Dec 16;123:106081. doi: 10.1016/j.ebiom.2025.106081 (PMC12768872; doi:10.1016/j.ebiom.2025.106081)
Supplement: Supplementary Figure S2 [file mmc2.docx]

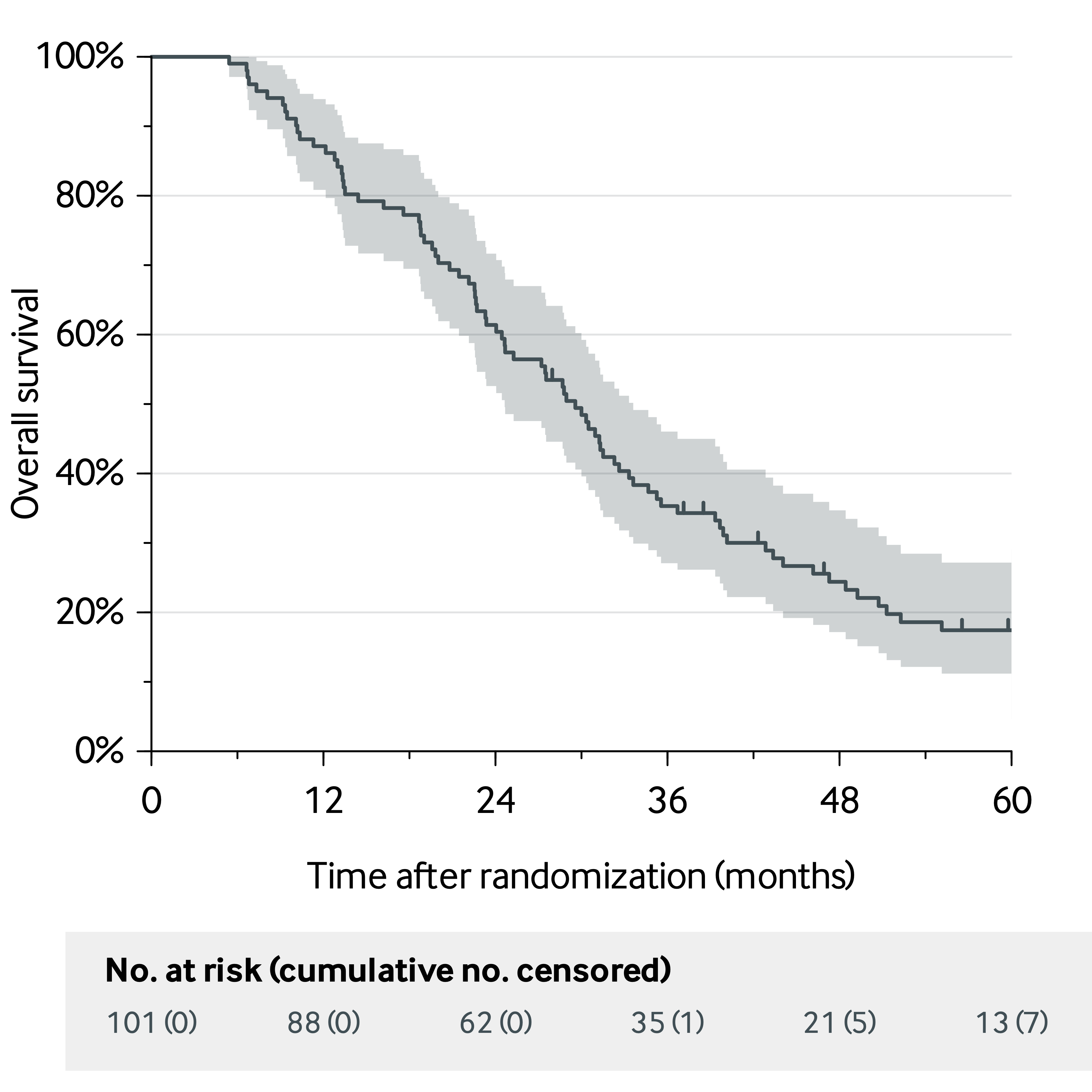


**Suppl. Figure 2.** Kaplan-Meier curve for all patients. The table below shows the number of patients at risk and the cumulative number of censored patients.
